# Screening of biomarkers and machine learning prediction in traumatic brain injury

**DOI:** 10.3389/fneur.2026.1847549

**Published:** 2026-05-25

**Authors:** Qianqian Chi, Yu Yin, Yuewen Song, Jianfang Li, Guoxing Xu, Zuncheng Zheng, Xiaoyu Wang, Xiuqiong Chen, Xiaojing Zhang, Lixin Zhang, Xiaolan Zhou, Zhe Li, Ziying Zhang, Rong Xu, Qian Zhong, Ling Liu, Lingxi Yin, Xiangming Ye, Xiao Lu, Xiao Lu, Qian Yu, Linghui Dong, Hao Zhang

**Affiliations:** 1 School of Rehabilitation, Capital Medical University, Beijing, China; 2 Department of Neurological Rehabilitation, Beijing Bo'ai Hospital, China Rehabilitation Research Center, Beijing, China; 3 Department of Rehabilitation Medicine, Hebei General Hospital, Shijiazhuang, Hebei Province, China; 4 Department of Rehabilitation Medicine, The First Hospital of Jilin University, Changchun, Jilin Province, China; 5 The Affiliated Taian City Central Hospital of Qingdao University, Taian, Shandong Province, China; 6 Department of Rehabilitation Medicine, Jiangbin Hospital of Guangxi Zhuang Autonomous Region, Nanning, Guangxi, China; 7 Department of Rehabilitation, Shengjing Hospital of China Medical University, Shenyang, Liaoning Province, China; 8 Department of Rehabilitation Medicine, The Fifth Affiliated Hospital of Zhengzhou University, Zhengzhou, Henan Province, China; 9 Department of Rehabilitation Medicine, Nanjing Drum Tower Hospital, Clinical College of Nanjing Medical University, Nanjing, Jiangsu Province, China; 10 Rehabilitation Medicine Center, West China Hospital, Sichuan University, Chengdu, Sichuan Province, China; 11 Center for Rehabilitation Medicine, Rehabilitation & Sports Medicine Research Institute of Zhejiang Province, Department of Rehabilitation Medicine, Zhejiang Provincial People's Hospital, Affiliated People's Hospital, Hangzhou Medical College, Hangzhou, Zhejiang, China; 12 Department of Rehabilitation Medicine, Guangdong Sanjiu Brain Hospital, Guangzhou, Guangdong Province, China; 13 Department of Rehabilitation Medicine, The First Affiliated Hospital of Nanjing Medical University, Nanjing, Jiangsu Province, China; 14 Department of Rehabilitation Medicine, Sichuan Academy of Medical Sciences & Sichuan Provincial People's Hospital, Chengdu, Sichuan Province, China; 15 China Rehabilitation Research Center, Beijing, China

**Keywords:** biomarker, cognitive function, machine learning, neurorehabilitation, traumatic brain injury

## Abstract

**Background:**

Traumatic brain injury (TBI) is a major cause of disability and mortality worldwide. Accessible blood and hormonal biomarkers may be related to injury severity and cognitive status in patients undergoing neurorehabilitation. This study used explainable machine learning to explore the associations of routine blood parameters and hormones with radiological injury severity and cognitive status in TBI.

**Methods:**

In this prospective cross-sectional multicenter study, 154 patients with TBI were enrolled. Blood samples were collected within 1 week after admission to assess routine hematological indices, liver and renal function, blood lipids, and hormone levels. Injury severity was evaluated using the Helsinki CT Score, and cognitive status was assessed using the mini-mental state examination (MMSE). After preprocessing, data were split into training and validation sets at a ratio of 7:3. LASSO regression was used for feature selection, and six machine learning models were developed. Model performance was evaluated using *R*^2^, mean squared error, and mean absolute error. SHapley Additive exPlanations were used for interpretation.

**Results:**

LASSO identified eight features for the Helsinki CT Score and four for MMSE. Random forest performed best for the Helsinki CT Score (validation *R*^2^ = 0.06), whereas CatBoost performed best for MMSE (validation *R*^2^ = 0.103). SHAP analysis indicated that IGF-1 was an important feature in both models. IGF-1 showed a possible nonlinear association with both outcomes.

**Conclusion:**

Routine blood and hormonal biomarkers, particularly IGF-1, may be associated with radiological injury severity and cognitive status in TBI. These findings are exploratory and require validation in larger longitudinal studies.

**Clinical trial registration:**

Chinese Clinical Trial Registry: ChiCTR2300072902. Medical Research Registration Number: MR-11-23-023826.

## Introduction

Traumatic brain injury (TBI) affects an estimated 50–60 million individuals globally each year, contributing to substantial morbidity, mortality, and an economic burden exceeding US$400 billion annually (1). As a leading cause of disability, particularly among young adults, TBI not only disrupts neurological function but also imposes profound societal costs through lost productivity, long-term care needs, and reduced quality of life. With rising incidences linked to traffic accidents, falls, sports-related injuries, and military exposures, understanding the multifaceted factors that modulate injury severity and cognitive outcomes is imperative for developing targeted clinical interventions, personalized rehabilitation strategies, and effective public health policies that mitigate long-term societal impacts.

TBI encompasses a broad spectrum of insults to the brain, ranging from mild concussions to severe diffuse axonal injuries, and initiates intricate pathophysiological cascades involving neuroinflammation, oxidative stress, mitochondrial dysfunction, and neuroendocrine dysregulation. These mechanisms frequently result in enduring cognitive impairments, including deficits in memory, attention, executive function, and processing speed, which hinder daily activities, vocational reintegration, and overall quality of life. Accessible biomarkers from peripheral blood, such as routine hematological parameters including hemoglobin (Hb) and hematocrit (Hct), liver enzymes like aspartate aminotransferase (AST), and proteins such as total protein (TP), serve as surrogates for systemic inflammatory and metabolic responses to brain trauma. Hormonal markers, notably insulin-like growth factor-1 (IGF-1), along with thyroid hormones (e.g., T4, fT3) and reproductive hormones (e.g., luteinizing hormone (LH), progesterone (P)), contribute to neuroprotection, synaptic plasticity, and metabolic homeostasis in the post-injury environment.

Previous investigations have underscored the prognostic significance of these biomarkers in TBI cohorts. For example, diminished IGF-1 levels have been associated with heightened symptom severity, depressive symptoms, and anxiety in adult patients with TBI (2). Preclinical rodent models have shown that IGF-1 administration mitigates hippocampal neurodegeneration, ameliorates gut-brain axis perturbations, and enhances behavioral outcomes following controlled cortical impact (3). Large-scale biobank studies further identify IGF-1, AST, and related markers as predictors of long-term functional recovery, emphasizing their role in bridging endocrine and neurological domains. Similarly, hematological alterations, such as reduced Hb and elevated AST, correlate with coagulopathy and secondary brain injury in acute TBI phases (4).

Despite the established roles of biomarkers like IGF-1, Hb, AST, TP, and hormonal factors in TBI, their complex, nonlinear associations with injury severity and cognitive function have not been fully elucidated. Conventional statistical approaches, such as linear regression, frequently fail to capture intricate interactions, threshold effects, and non-monotonic relationships, resulting in oversimplified models that overlook subtle biomarker dynamics (5). For instance, while lower IGF-1 concentrations generally link to adverse outcomes, inconsistencies across studies—attributable to variations in patient demographics, injury mechanisms, or analytical assumptions—reveal unresolved discrepancies in dose-response patterns (6). The advent of machine learning (ML) has revolutionized TBI prognostication by assimilating heterogeneous data modalities, including clinical scores, biomarkers, and neuroimaging, to outperform conventional logistic regression in outcome prediction (7). Ensemble methods like random forest (RF) and gradient boosting algorithms (e.g., XGBoost, CatBoost) excel in handling nonlinearities and interactions, while explainable artificial intelligence (XAI) tools, such as SHapley Additive exPlanations (SHAP), provide interpretable insights into feature importance and dependencies (8). These interdisciplinary advancements integrate neurology, endocrinology, computational biology, and data science, fostering applications in precision medicine, such as early risk stratification in emergency settings and hormone-based therapies to promote neuroregeneration.

The present study aimed to describe and explain the effects of key blood and hormonal factors on TBI using explainable machine learning. We hypothesized that routine hematological parameters and hormonal parameters, including IGF-1 and Hb, can serve as biomarkers and exhibit nonlinear and interactive relationships with injury severity, cognitive function. To achieve this, we performed feature selection via LASSO regression, then optimized ensemble ML models through cross-validation. Additionally, we conducted SHAP analysis to elucidate feature contributions, dependencies, and partial effects, thereby uncovering novel mechanisms underlying injury progression and recovery and providing references for clinical interventions.

## Methods

### Study design and participants

This prospective cross-sectional study enrolled 154 patients with traumatic brain injury (TBI) from 14 medical centers. The diagnosis of TBI was confirmed by cranial computed tomography (CT) or cranial magnetic resonance imaging (MRI). All patients were aged 18–65 years, with a disease duration of 1 month to 24 months. Patients were excluded if they had disturbance of consciousness, unstable vital signs, severe circulatory system diseases, renal insufficiency, epileptic seizures within 1 month before enrollment, or growth hormone use within 1 year before enrollment.

All patients underwent blood sample collection within 1 week after admission, along with imaging and cognitive assessments. All participating centers followed a common study protocol for patient enrollment, blood collection, biomarker testing, and outcome assessment. Helsinki CT Score and MMSE were evaluated according to unified criteria, and data collection was standardized across centers. This study was conducted in accordance with the Declaration of Helsinki and was approved by the Medical Ethics Committee of Beijing Bo'ai Hospital, China Rehabilitation Research Center (Ethics Approval No.: 2021-141-2). Written informed consent was obtained from all participants or their legal representatives.

### Data collection and biomarkers

Venous blood samples were obtained from patients for the measurement of routine blood parameters and hormone levels. Specific biomarkers included: white blood cell count (WBC), red blood cell count (RBC), hemoglobin concentration (Hb), hematocrit (Hct), mean corpuscular hemoglobin (MCH), mean corpuscular hemoglobin concentration (MCHC), platelet count (PLT), neutrophil percentage (Neut%), lymphocyte percentage (Lymph%), monocyte percentage (Mono%), eosinophil percentage (Eos%), basophil percentage (Baso%), alanine transaminase (ALT), aspartate aminotransferase (AST), total protein (TP), albumin (Alb), alkaline phosphatase (ALP), total bilirubin (TBil), direct bilirubin (DBil), creatinine (Cr), urea, uric acid (UA), glucose (Glu), cholesterol (Chol), triglyceride (TG), low-density lipoprotein cholesterol (LDL-C), high-density lipoprotein cholesterol (HDL-C), prothrombin time (PT), activated partial thromboplastin time (APTT), thrombin time (TT), and fibrinogen (Fib). Hormonal markers included growth hormone (GH), insulin-like growth factor-1 (IGF-1), cortisol, adrenocorticotropic hormone (ACTH), triiodothyronine (T3), thyroxine (T4), free triiodothyronine (fT3), free thyroxine (fT4), thyroid-stimulating hormone (TSH), follicle-stimulating hormone (FSH), luteinizing hormone (LH), testosterone (T), estradiol (E2), and progesterone (P). All assays were performed using standardized laboratory protocols under uniform conditions.

### Outcome measures

Injury severity was evaluated using the Helsinki CT Score, a radiological grading system based on cranial CT features including cerebral edema, hematoma volume, and ventricular compression. The score ranges from −3 to 14, with higher values indicating more severe injury. Cognitive function was assessed using the Mini-Mental State Examination (MMSE), with scores ranging from 0 to 30; higher scores indicate better cognitive performance. All assessments were performed within 1 week after admission by trained neurologists who conducted evaluations independently.

### Data preprocessing and machine learning analysis

Data preprocessing included outlier detection using the interquartile range (IQR) method with a threshold of 1.5 × IQR. To reduce the risk of information leakage, the dataset was first randomly divided into training and validation sets at a ratio of 7:3, and missing values were then imputed separately after data splitting. Missing-data imputation was performed using LightGBM (LGBM), with the training set and validation set processed independently within the predefined preprocessing workflow. This strategy was adopted to improve methodological transparency and to avoid introducing information from the validation set into the model development process. Least absolute shrinkage and selection operator (LASSO) regression was applied for feature selection, with λ chosen to minimize cross-validation error.

Six machine learning models were constructed for the outcome variables of Helsinki CT Score and MMSE score: k-nearest neighbor (KNN), random forest (RF), extreme gradient boosting (XGBoost), CatBoost, LightGBM (LGBM), and multilayer perceptron (MLP). Model optimization was performed using 5-fold cross-validation, and performance was evaluated using the coefficient of determination (*R*^2^), mean squared error (MSE), and mean absolute error (MAE). The model with the highest validation-set *R*^2^ was selected as the optimal model.

The SHapley Additive exPlanations (SHAP) framework was then used to interpret the optimal model, including feature importance calculation, SHAP summary beeswarm plots, scatter plots, and partial dependence plots (PDP). All analyses were conducted in Python 3.8 with scikit-learn, XGBoost, CatBoost, LightGBM, and SHAP packages. Statistical significance was defined as *P* < 0.05.

## Results

A total of 154 patients with traumatic brain injury (TBI) were initially enrolled. 10 patients were excluded due to incomplete data, resulting in 144 patients included in the final statistical analysis. The baseline characteristics of the study population are summarized as follows: the mean age was 43.9 ± 13.6 years (median, 44 years); 116 patients were male (mean age, 44.7 ± 13.3 years) and 28 were female (mean age, 40.4 ± 14.0 years); the mean body mass index (BMI) was 23.5 ± 4.6 kg/m^2^. With respect to disease duration, 73 patients had a disease course of 1–3 months, 34 patients had 4–6 months, and 37 patients had 7–24 months.

### Prediction models based on the Helsinki CT score

**Figure 1:**
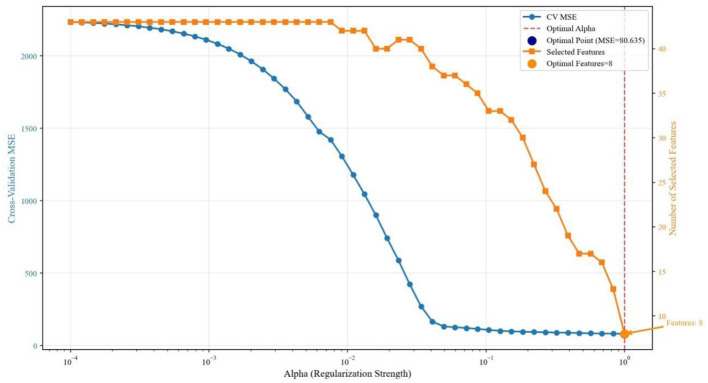
LASSO regression: alpha vs. number of features vs. model performance.

Predictive models were constructed using the Helsinki CT Score as the outcome variable. LASSO regression-based feature selection identified an optimal set of eight features for model development (Figure 1), including hemoglobin (Hb), aspartate aminotransferase (AST), total protein (TP), insulin-like growth factor-1 (IGF-1), thyroxine (T4), free triiodothyronine (fT3), luteinizing hormone (LH), and progesterone (P).

**Figure 2:**
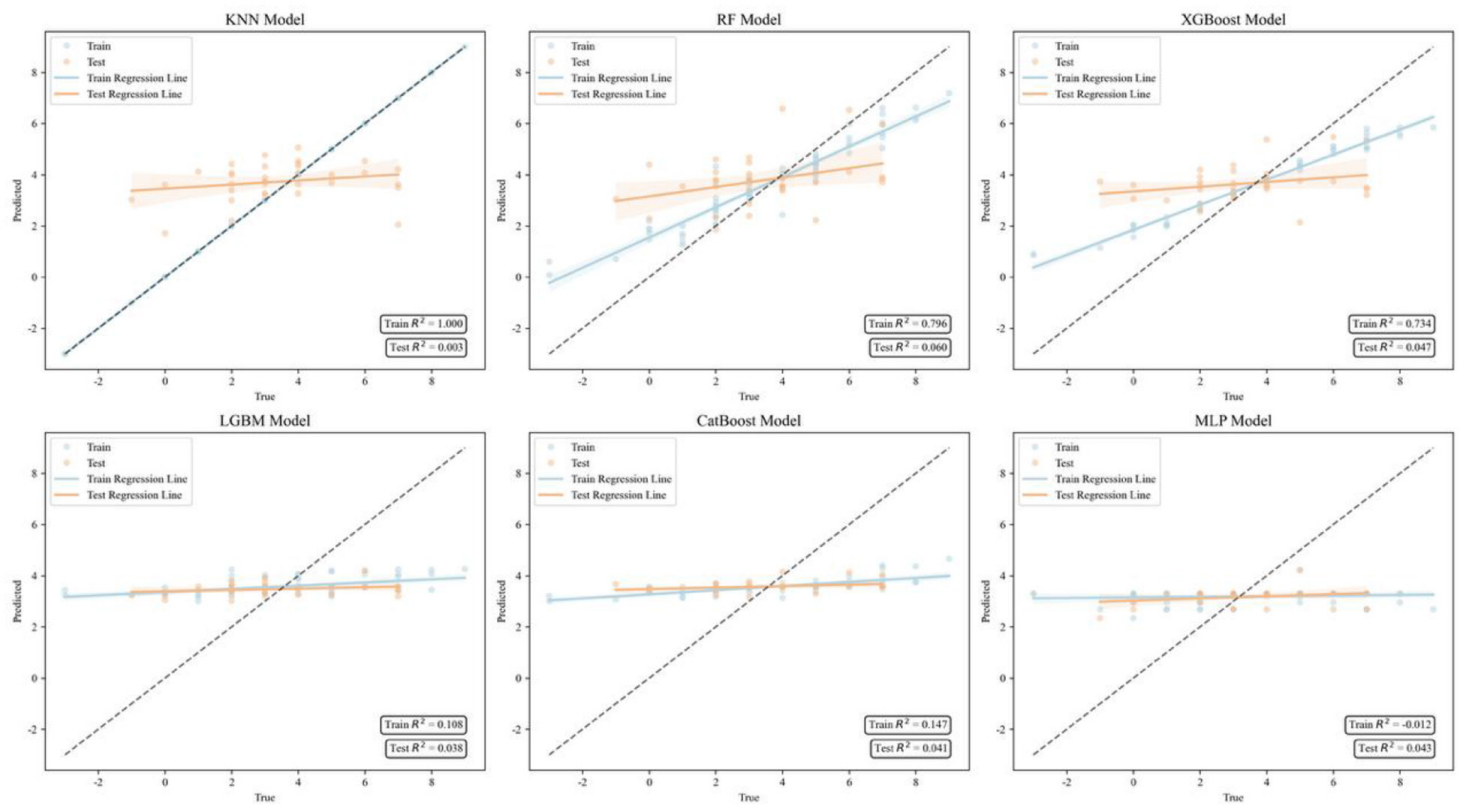
Scatter plots and *R*^2^ of six models in training and validation sets.

Six machine learning predictive models were established, and the coefficient of determination (*R*^2^) values of these six models under optimized hyperparameters are presented in Figure 2. Among these models, the random forest (RF) model achieved the highest *R*^2^ value (0.06) in the validation set (Figure 2).

**Figure 3:**
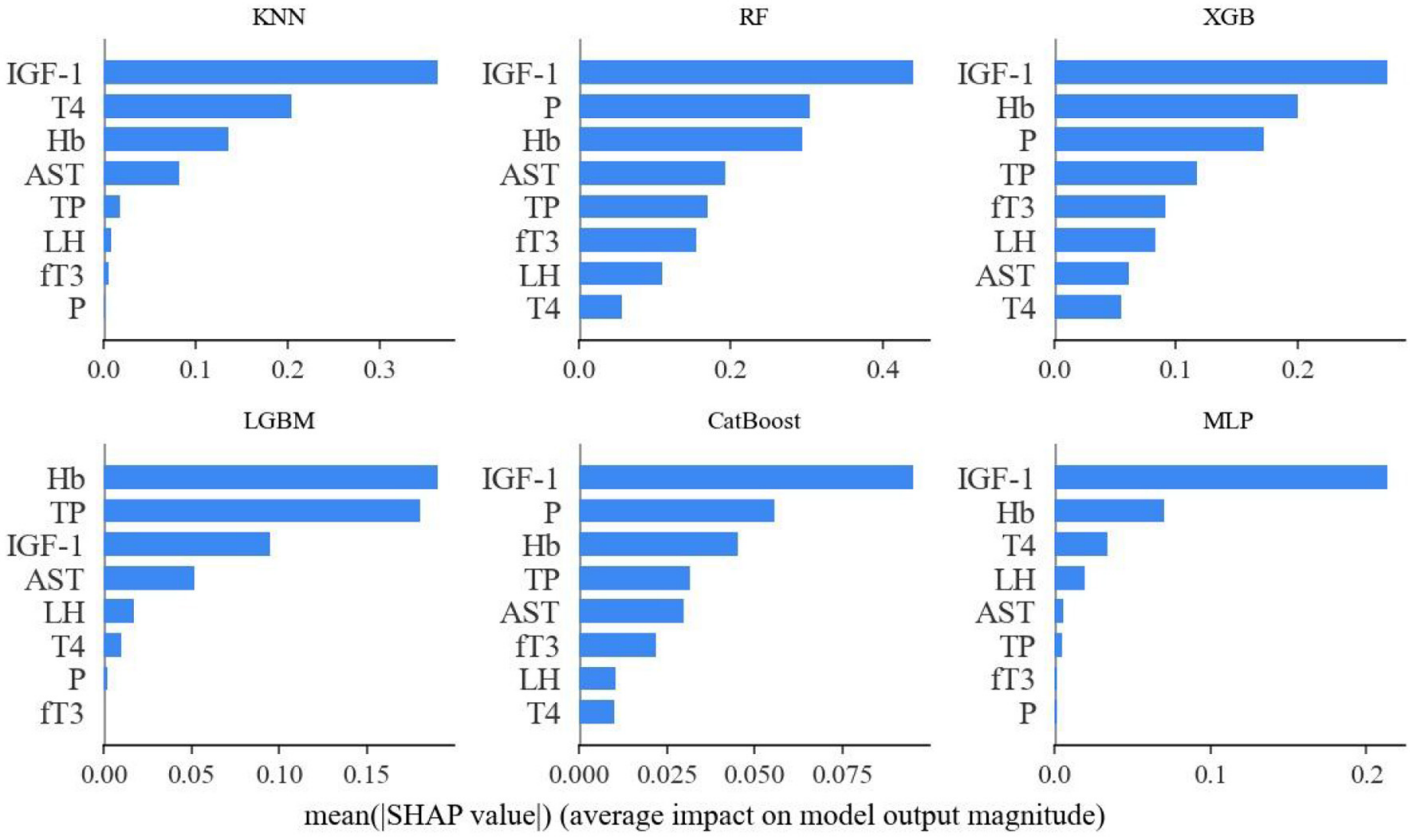
SHAP sorted feature importance of six models in validation sets.

**Figure 4:**
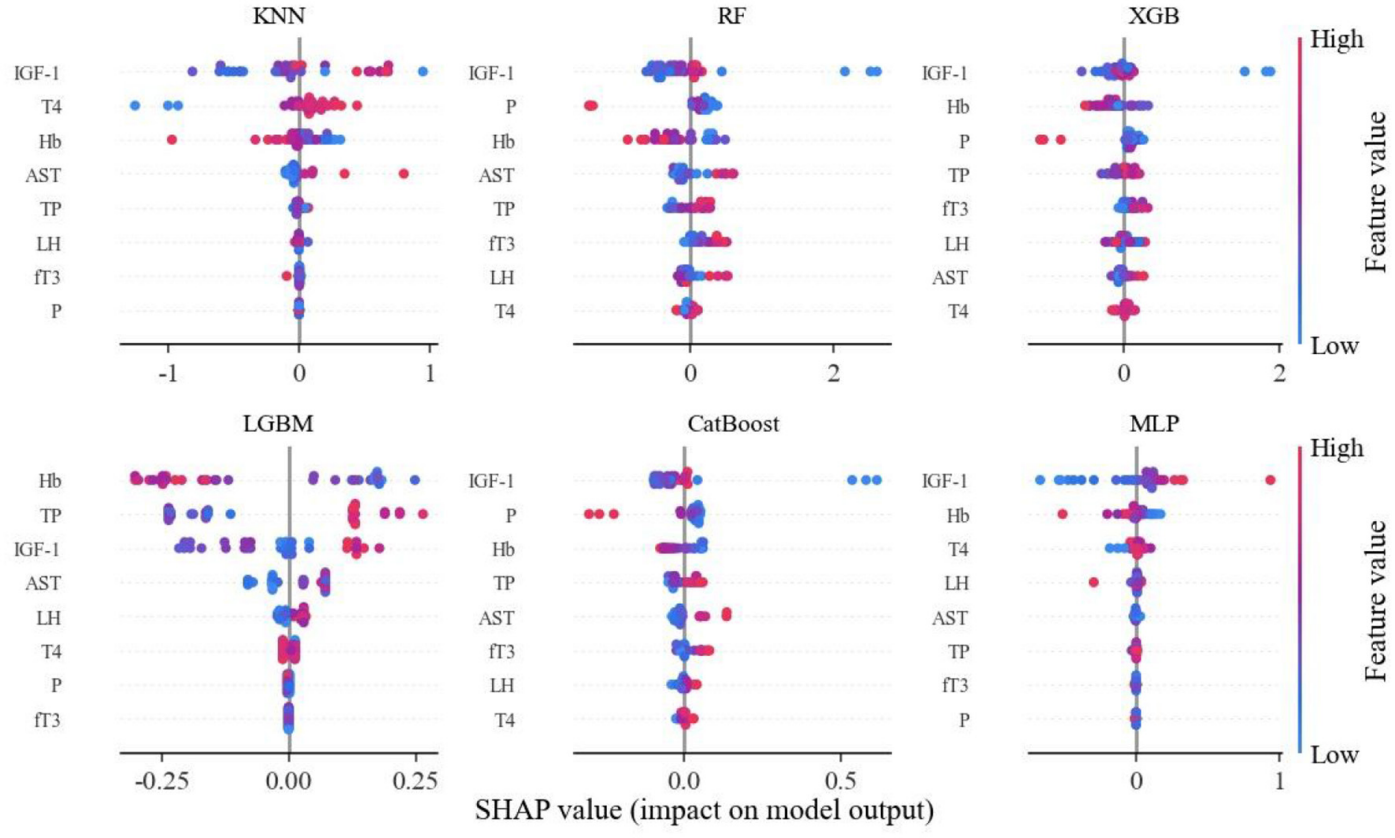
SHAP feature importance analysis of six models in validation sets.

The SHapley Additive exPlanations (SHAP) framework was employed to interpret the impact of each feature on the Helsinki CT Score. IGF-1 was identified as the most important predictor of the CT score in 5 out of the 6 constructed models, including the optimal RF model (Figures 3, 4).

**Figure 5:**
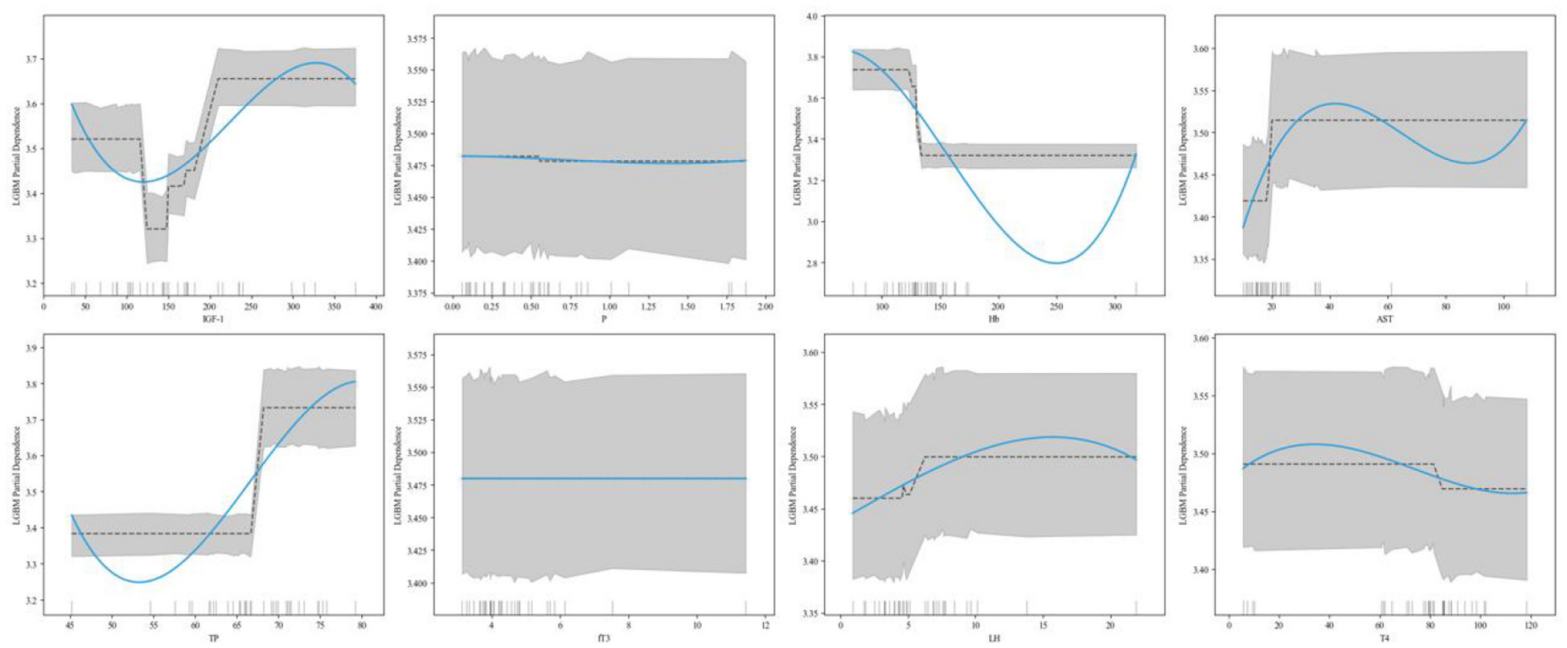
Scatter plots of features in the optimal RF model.

**Figure 6:**
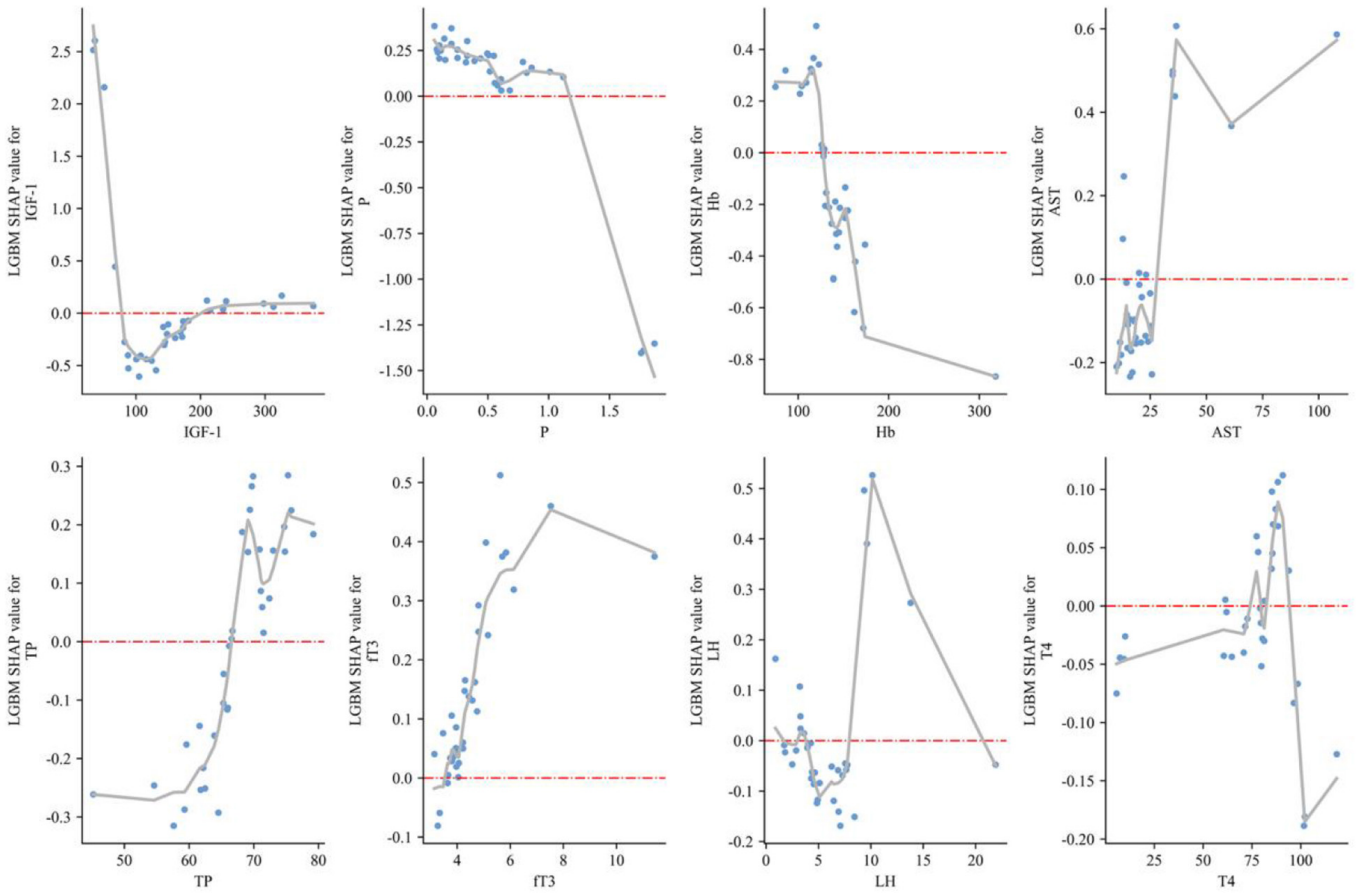
Partial dependence plots of features in the optimal RF model.

The optimal RF model was selected for further analyses, and scatter plots (Figure 5) and partial dependence plots (PDPs, Figure 6) were generated to illustrate the relationship between each feature and the CT score. With the elevation of IGF-1, TP, and Hb levels, the CT score exhibited a trend of initial decrease followed by an increase. In contrast, the CT score first increased and then decreased with rising AST levels, indicating a significant nonlinear relationship. Additionally, the CT score showed an overall decreasing trend with the increase in progesterone (P) levels.

### Prediction models based on the MMSE score

**Figure 7:**
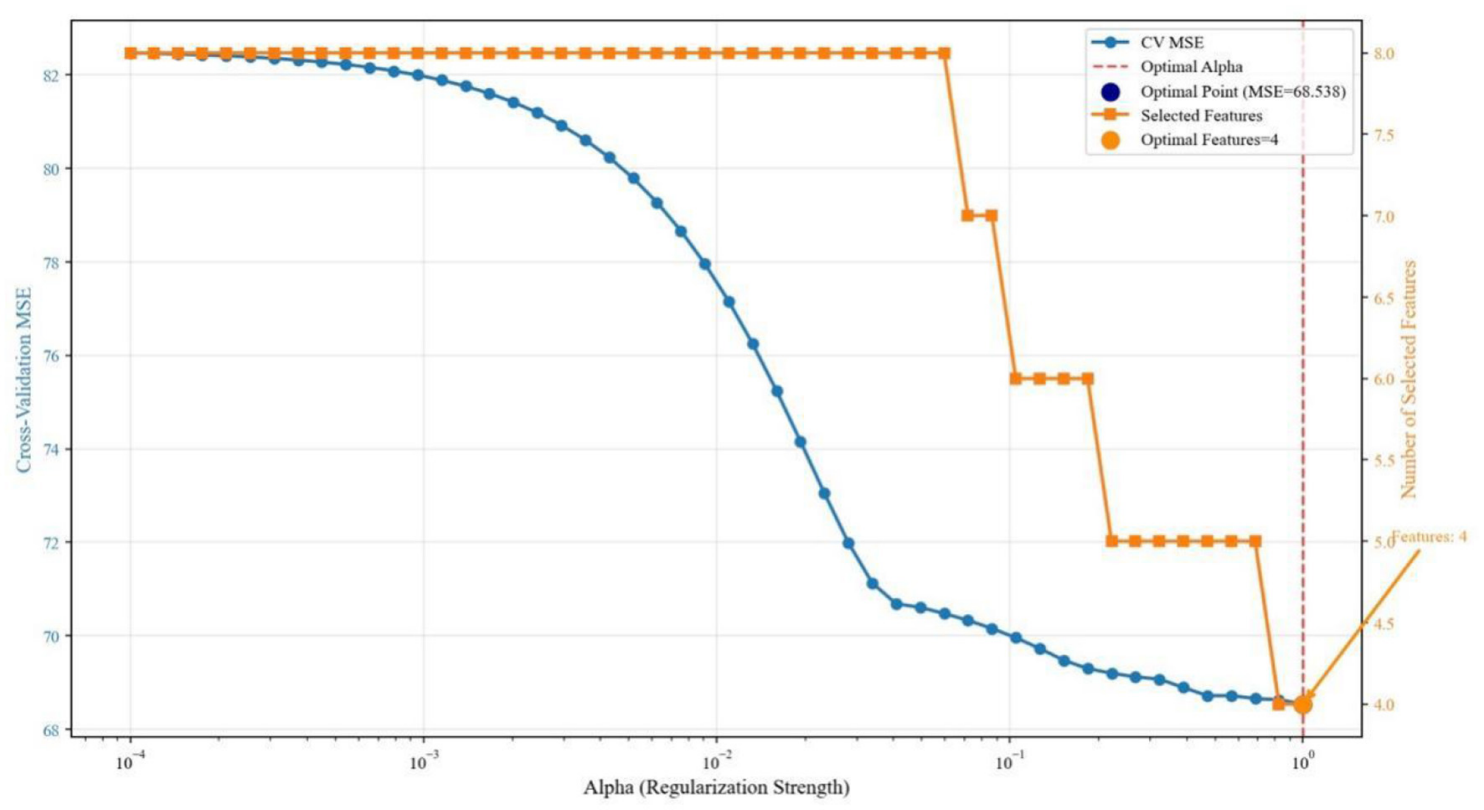
LASSO regression: alpha vs. number of features vs. model performance.

Predictive models were constructed with the MMSE score as the outcome. LASSO feature selection identified four optimal features for modeling (Figure 7): hemoglobin (Hb), hematocrit (Hct), creatinine (Cr), and insulin-like growth factor-1 (IGF-1).

**Figure 8:**
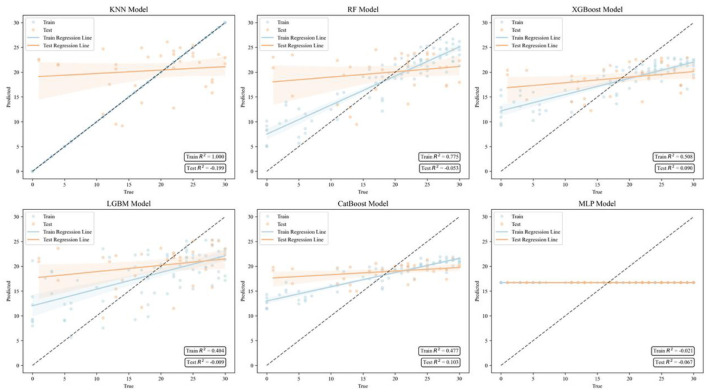
Scatter plots and *R*^2^ of six models in training and validation sets.

Six models were established; their *R*^2^ values under optimized hyperparameters are shown in Figure 8. The CatBoost model had the highest validation-set *R*^2^ (0.103, Figure 8).

**Figure 9:**
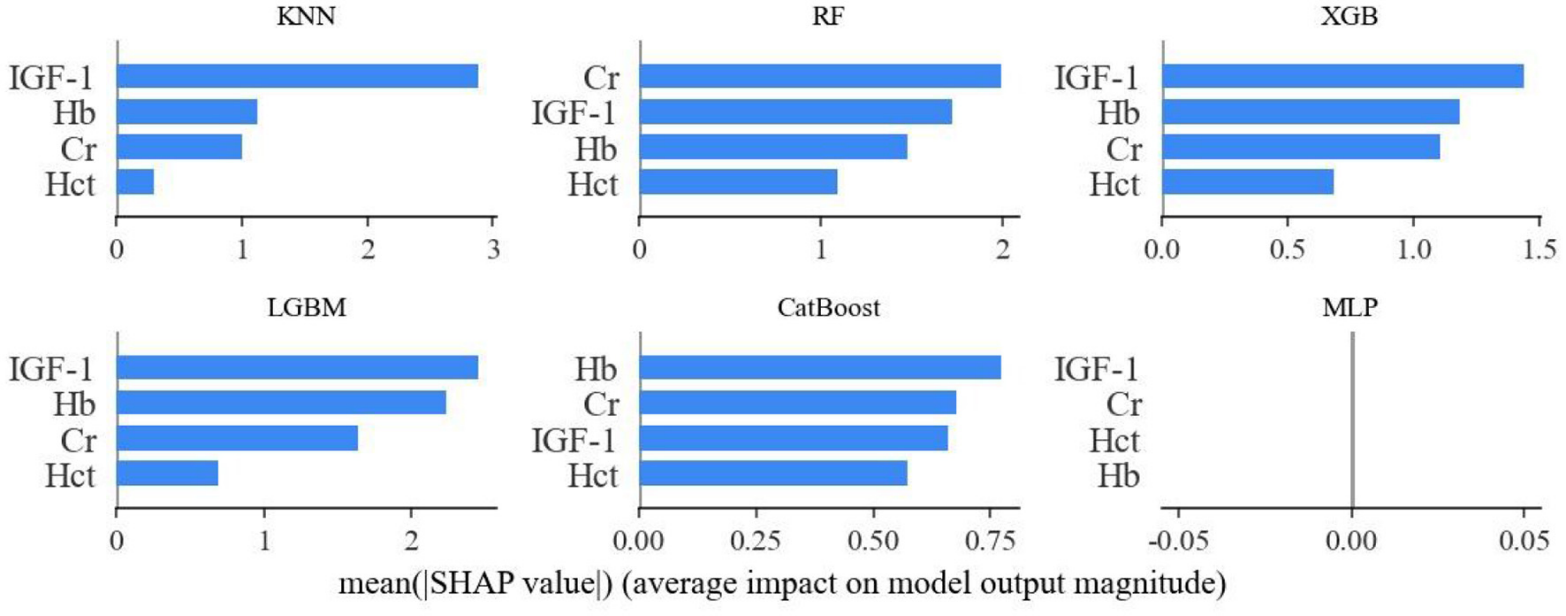
SHAP sorted feature importance of six models in validation sets.

**Figure 10:**
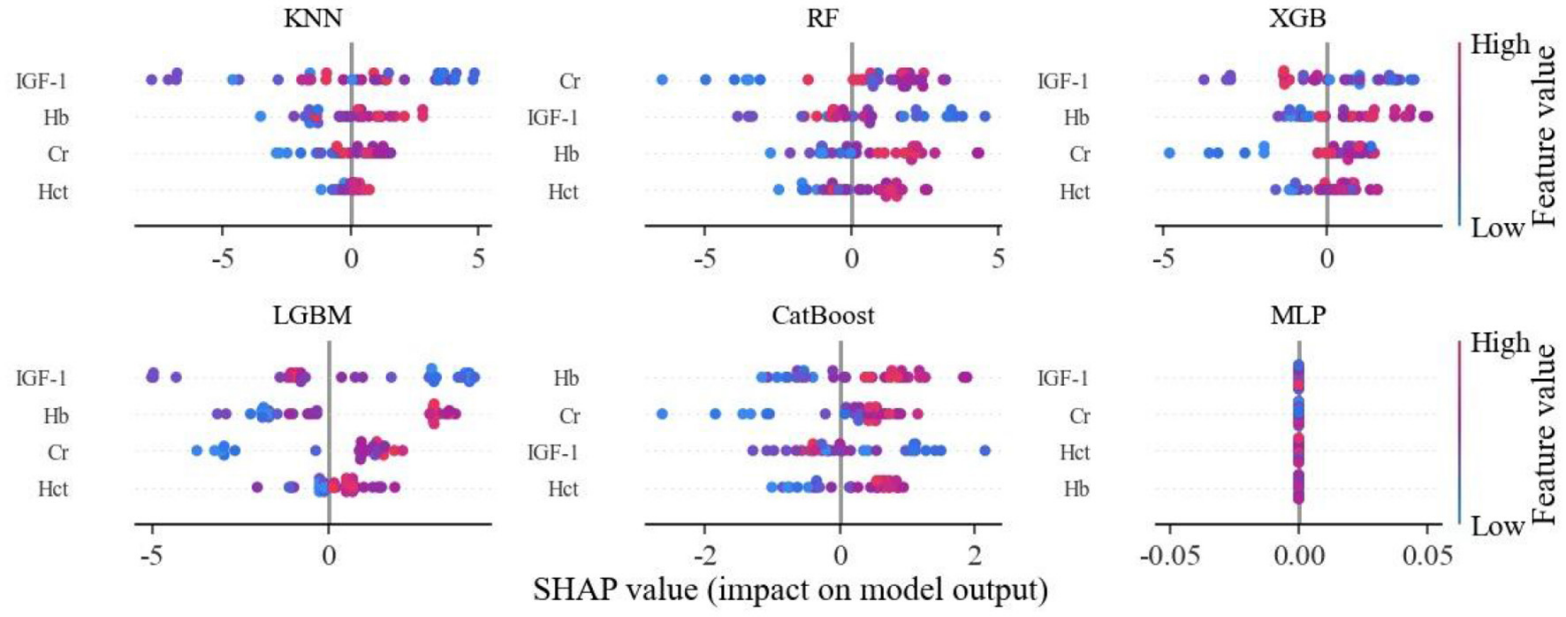
SHAP feature importance analysis of six models in validation sets.

SHAP analysis interpreted feature contributions to MMSE scores. IGF-1 was the most important predictor in 3 of 6 models, but Hb was the top feature in the optimal CatBoost model (Figures 9, 10).

**Figure 11:**
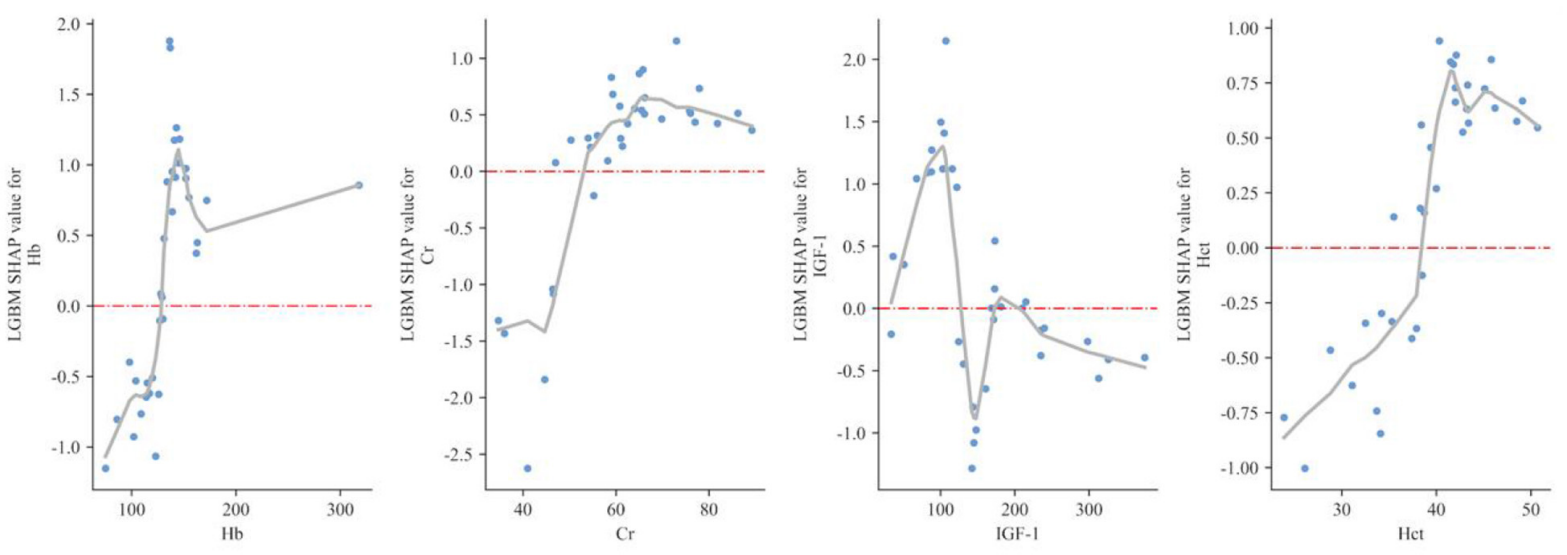
Scatter plots of features in the optimal CatBoost model.

**Figure 12:**
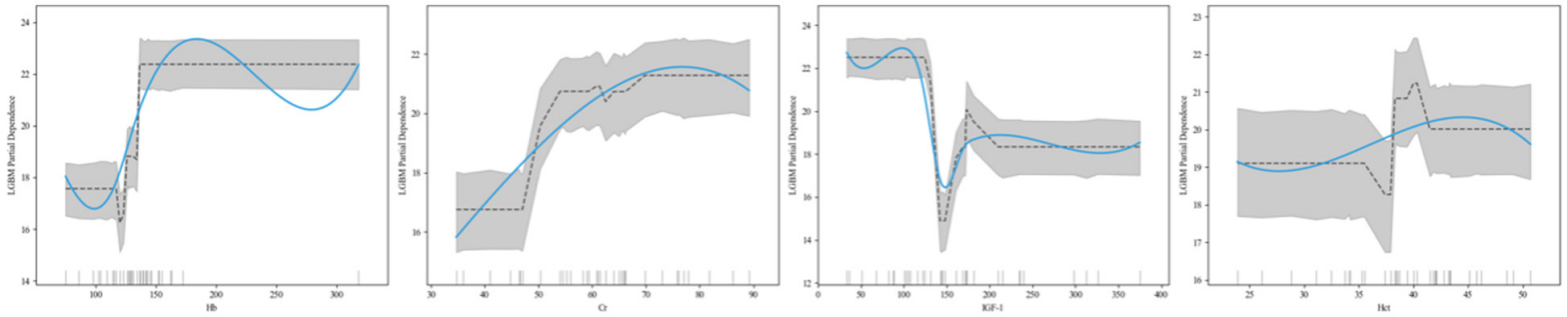
Partial dependence plots of features in the optimal CatBoost model.

Further analysis of the CatBoost model (scatter plots, Figure 11; partial dependence plots, Figure 12) showed that higher Hb, Hct, and Cr were associated with higher MMSE scores. The MMSE score increased initially, then decreased, and rose again with increasing IGF-1.

## Discussion

This study used explainable machine learning to explore blood and hormonal biomarkers associated with radiological injury severity and cognitive status in patients with TBI undergoing neurorehabilitation. Among the identified features, IGF-1 emerged as an important variable in models for both the Helsinki CT Score and MMSE, together with contributions from Hb, AST, TP, Hct, and Cr. Overall, these findings suggest that accessible blood and hormonal indicators may contain clinically relevant information related to injury severity and cognitive status in this population.

One clinically relevant observation is the prominence of IGF-1 across both models. This finding is biologically plausible because post-traumatic pituitary dysfunction is common after TBI and may influence both recovery trajectories and cognitive performance during rehabilitation (9). Growth hormone deficiency has been described as one of the most frequent chronic endocrine abnormalities after TBI, which provides a broader framework for interpreting an IGF-1 signal in this setting (10). At the same time, serum IGF-1 should not be treated as a direct surrogate for post-traumatic growth hormone deficiency. In a dedicated diagnostic study, baseline IGF-1 had limited value as a screening test for growth hormone deficiency after TBI or sport-related concussion (11). Our results therefore support a more cautious interpretation. IGF-1 may reflect a complex neuroendocrine state relevant to injury and recovery, but it is unlikely to function as a simple diagnostic threshold on its own.

Experimental work also supports a mechanistic link between IGF-1 and post-traumatic repair. In animal models, local overexpression of IGF-1 enhances hippocampal neurogenesis and restores dendritic structure in immature neurons after TBI (12). Subsequent work further showed that IGF-1-driven hippocampal remodeling was accompanied by better spatial memory in experimental TBI (13). These data are consistent with the possibility that the nonlinear pattern seen in our SHAP and partial dependence analyses may reflect the competing effects of deficiency, compensatory activation, and systemic illness. However, the present study was not designed to define a therapeutic range, and the apparent turning points in the model should not be interpreted as evidence of a clinically actionable threshold.

Available clinical literature points in the same direction but remains limited. A systematic review reported that growth hormone treatment initiated in the chronic phase after TBI may improve aspects of processing speed, memory, mood, and quality of life, although the underlying studies were small and methodologically heterogeneous (14). A recent scoping review reached a similar conclusion and emphasized the need for larger controlled studies before hormone replacement can be generalized beyond carefully selected patients (15). Taken together, these reports suggest that the IGF-1 axis is relevant to rehabilitation outcomes, but they do not justify treatment decisions based solely on a single serum measurement.

Hematological variables were also informative in our models, especially Hb and Hct in relation to MMSE. This is in line with prior work showing that lower hemoglobin is associated with worse outcome after TBI (16). More recent multicenter data from CENTER-TBI likewise showed that anemia during the 1st week of intensive care was independently associated with unfavorable neurological outcome and mortality (17). Severe anemia also appears to be common after acute moderate to severe TBI, often developing within the first 48 h after trauma (18). A likely explanation is that reduced oxygen-carrying capacity may aggravate secondary cerebral hypoxia in a brain that is already metabolically vulnerable. Even so, the translational implications remain uncertain, because transfusion thresholds in TBI continue to be debated and liberal transfusion has not consistently improved outcomes (19, 20). Our findings therefore support the relevance of Hb and Hct as markers of systemic physiological reserve, rather than as direct treatment targets in isolation.

The appearance of TP and AST in the Helsinki CT model may also be clinically meaningful, although these markers are less specific. Total protein may partly reflect nutritional state, inflammatory burden, hemodilution, or altered hepatic synthesis. In related literature, lower serum albumin has been associated with poorer outcome after TBI (21). In pediatric moderate to severe TBI, admission albumin also outperformed hemoglobin in predicting mortality and short-term resource use (22). Because total protein is a broader and less specific measure than albumin, its contribution in our model should be viewed as hypothesis-generating. AST may similarly reflect systemic tissue injury or metabolic stress rather than a brain-specific pathway. These observations support the idea that part of the biomarker signal identified by machine learning may represent extra-cerebral physiological disturbance that still bears on brain recovery.

The hormonal variables T4, fT3, LH, and progesterone identified during feature selection are also notable. Thyroid dysfunction after TBI has been linked to neurological and functional outcomes in the subacute rehabilitation phase (23). Persistent pituitary hormone abnormalities have likewise been documented months after injury, which reinforces the relevance of endocrine surveillance in selected patients undergoing rehabilitation (25). The inverse association between progesterone and radiological severity in our model should be interpreted with particular caution. Endogenous progesterone may be a marker of broader endocrine status, but it should not be conflated with treatment efficacy. Large phase III trial data did not show clinical benefit from progesterone therapy in severe TBI (24).

From a methodological perspective, our results highlight both the value and the limits of explainable machine learning in biomarker studies. Conventional regression can miss interaction effects and non-monotonic associations, whereas SHAP-based interpretation can make such patterns visible at the individual feature level (5, 8). That advantage is relevant in TBI, where endocrine, inflammatory, hematological, and metabolic responses evolve together rather than along a single pathway. At the same time, the low validation-set *R*^2^ values in the present study indicate that only a small proportion of between-patient variance was captured. This likely reflects the clinical heterogeneity of the cohort, the restricted sample size, and the fact that outcome after TBI is determined by many factors not included here, such as premorbid reserve, injury biomechanics, lesion distribution, treatment intensity, and rehabilitation exposure.

Our results fit that literature. The value of the present analysis lies more in hypothesis generation and feature discovery than in predictive performance. Taken together, this study suggests that routine blood and hormonal biomarkers, particularly IGF-1, may be associated with radiological injury severity and global cognitive status in TBI. The use of explainable machine learning provided an interpretable framework for identifying potentially relevant features and visualizing possible relationship patterns. These findings offer exploratory evidence that may help guide future studies on accessible biomarkers in TBI neurorehabilitation.

### Limitations

This study has several limitations. First, the sample size was modest, and the wide disease-duration range (1–24 months) may have introduced clinical heterogeneity, as biomarker profiles, endocrine status, imaging findings, and cognitive performance can vary across different stages of TBI. Second, although all participating centers followed a common study protocol, residual inter-center differences in testing platforms, implementation details, and clinical practice may still have influenced the results. Third, this study was designed to focus on routine blood and hormonal biomarkers rather than to construct a fully comprehensive clinical model; therefore, some potentially important clinical confounders were not incorporated into the modeling process. In addition, given the limited sample size, the use of multiple machine learning models and a single train-validation split may have affected model stability, and the relatively low validation-set *R*^2^ values indicate limited predictive performance. Finally, the cross-sectional design precludes causal inference, and the observed associations, including the suggested nonlinear patterns, should be regarded as exploratory. Larger longitudinal studies with more homogeneous populations and more comprehensive clinical adjustment are needed for further validation.

## Conclusion

This study suggests that routine blood and hormonal biomarkers, particularly IGF-1, may be associated with radiological injury severity and cognitive status in patients with TBI undergoing neurorehabilitation. Explainable machine learning provided an interpretable approach for identifying potentially relevant features and visualizing possible association patterns. These findings are exploratory and hypothesis-generating, and require further validation in larger studies with longitudinal follow-up.

## Data Availability

The datasets presented in this article are not readily available due to ethical restrictions, data are not available upon request without prior ethics approval. Requests to access the datasets should be directed to Hao Zhang, crrczh2020@163.com.
